# Assessment of Quality of Life in Head-and-Neck Oncologic Patients with Intraoral Soft-Tissue Defects Reconstructed with Buccinator Myomucosal Flap

**DOI:** 10.3390/jcm11247458

**Published:** 2022-12-15

**Authors:** Marc Agea Martínez, Raúl Antúnez-Conde, Carlos Navarro Cuéllar, Manuel Tousidonis Rial, Ignacio Navarro Cuéllar, Ana María López López, Dafne Gascón Alonso, Ángela Sada Urmeneta, José J. Zamorano-León

**Affiliations:** 1 Maxillofacial Surgery Department, Hospital General Universitario Gregorio Marañón, 28007 Madrid, Spain; 2 Public Health and Maternal & Child Health Department, School of Medicine, Universidad Complutense, 28040 Madrid, Spain

**Keywords:** quality of life, EORTC QLQ-C30, EORTC QLQ-H&N35, head and neck, buccinator flap, reconstructive surgery

## Abstract

The aim of this study is to evaluate the functional outcomes and quality of life (QoL) in oncologic patients with intraoral defects reconstructed with the buccinator myomucosal flap. A retrospective study was performed involving 39 patients with intraoral soft-tissue defects, reconstructed with a buccinator myomucosal flap during a six-year period. Patients completed the European Organization for Research and Treatment of Cancer questionnaires, the standard questionnaire (QLQ-C30) and the head-and-neck specific module (QLQ-H&N35). Thirty-nine patients with a mean age of 61.23 ± 15.80 years were included in the study. Thirty-three patients were diagnosed with an oncological condition (84.61%). Six patients (15.38%) developed orosinusal communication and underwent extensive debridement. The median global-health-status score was 79.27 and emotional performance was the lowest scoring, with a mean score of 76.93. As for the symptom items, the most outstanding were dental problems (33.33), oral opening (31.62) and dry mouth (37.61), followed by sticky saliva (24.79), problems with social eating (21.15) and pain (19.87). The most significant symptoms were radiotherapy-related adverse effects such as pain, fatigue, dental problems and dry mouth. Patients reconstructed with the buccinator myomucosal flap develop a good quality of life for all types of activities, and a correct function and aesthetics. Postoperative radiotherapy is associated with a poorer quality of life, and can lead to impairment of several symptoms such as swallowing, oral opening and dry mouth.

## 1. Introduction

The surgical management of oncologic patients may lead to intraoral soft-tissue defects that require immediate reconstruction to reestablish form and function. The ablative and reconstructive surgery can be challenging, and can be approached with different techniques, depending on the extension of the tumor, the nodal staging, and the involvement of other structures [1]. Small defects are usually reconstructed by primary closure or secondary-intention wound healing. Extensive or complex defects are usually reconstructed with free flaps, depending on patient morbidity and technical limitations. Medium-sized defects are usually reconstructed with local flaps that provide similar tissue, with low morbidity [1,2,3,4].

Disorders resulting from ablative and reconstructive surgery can significantly affect the quality of life in oncologic patients. Quality of life (QoL) is a wide and multidimensional concept that comprises many aspects of life: physiological, emotional and psychological [5]. It is considered a system that represents the individual’s general perception of well-being [6]. Concern in this area is now a key issue that can be reflected in increased research interest.

The aim of this study was to evaluate the functional outcomes and quality of life (QoL) in oncologic patients with intraoral soft-tissue defects, reconstructed with a buccinator myomucosal flap [6], by means of verified questionnaires. Therefore, the QLQ questionnaire of the European Organization for Research and Treatment of Cancer (EORTC) was implemented and analyzed. It is an integrated system for assessing patients’ health-related quality of life [7,8]. It includes a general module of 30 questions (QLQ-C30) and a specific module for the head-and-neck area with 35 questions (QLQ-H&N35) [9]. To date, this is the first study to report EORTC-verified QLQ questionnaires to assess the functionality, health and well-being among patients with intraoral soft-tissue reconstruction with the buccinator myomucosal flap.

## 2. Materials and Methods

A single-center retrospective study was designed to include 49 patients treated in the Oral and Maxillofacial Surgery Department at Gregorio Marañón General Hospital in Madrid, Spain, from January 2015 to September 2021. The study and review of the medical records and data collection, and the subsequent analysis of the data collected is endorsed by the Hospital Ethics Committee. Informed consent was obtained from all subjects involved in the study.

Inclusion criteria were: (1) reconstruction of intraoral defects with buccinator flap; (2) patients free of oncological disease or recovery from previous pathology, and follow-up of at least 6 months after successful treatment; (3) the interviewer was a different physician than the one who performed the usual follow-up.

Exclusion criteria were: (1) inability to understand or inability to complete the questionnaires; (2) failure to complete the questionnaires; (3) more than one local or regional surgical- procedure prior to surgical reconstruction with buccinator flap; (4) radiotherapy prior to surgical reconstruction. Ten patients were excluded: five patients refused to participate, three patients underwent previous radiotherapy and two patients had undergone two surgical procedures prior to surgical reconstruction.

### 2.1. Questionnaires

Since its first publication in 1993, the QLQ-C30 has been modified three times. Currently, version 3.0 of the QLQ-C30 and version 1.0 of the head-and-neck specific module (QLQ-H&N35) are implemented. The QLQ-C30 comprises five functioning scales (physical, role, emotional, cognitive and social functioning), nine symptom-scales (fatigue, nausea and vomiting, pain, dyspnea, insomnia, loss of appetite, constipation, diarrhea and economic difficulties) and a global-health-status scale. The first 28 questions provide four answer options measured on a Likert scale (not at all: 1, a little: 2, quite a lot: 3, a lot: 4), while the last two questions concern overall health, and are scored from 1 to 7 (with 1 being poor health and 7 being excellent health).

The QLQ-H&N35 module is designed to be a complement to the QLQ-C30 in order to increase the scope, sensitivity and specificity of the assessments. It includes 35 questions measuring symptoms and related problems in the head-and-neck area (pain, swallowing, coughing, dental problems, oral opening, dry mouth, sticky saliva, sensory problems, feeling sick, speech, social eating, social contact, sexuality, need for nutritional supplements or analgesics, and weight changes). The first 30 questions are scored according to a Likert scale, while the last five questions are answered in a dichotomous model (no: 1; yes: 2).

The scores of the questionnaires are calculated according to the instructions of the EORTC scoring manual. The score obtained for each item is a linear transformation from 0 to 100, whereby higher scores represent a higher level of response. Thus, high scores on symptom scales represent more symptomatology and a worse QoL, while high scores on functioning and global-health-scales represent a high QoL [7,8].

### 2.2. Reliability

The reliability of the questionnaires was evaluated by means of Cronbach’s alpha coefficient, obtaining a total score of 0.95, which is considered an excellent and internally-consistent result, with a value above 0.9.

### 2.3. Data Recording

The following sociodemographic and clinical data were included at the time of surgery: age, sex, comorbidity, smoking, alcohol consumption, primary diagnosis, and tumor-stage, according to the American Joint Committee on Cancer (AJCC) guidelines in the cases of oncologic disease, location and size of the defect, whether the defect included the resection of soft tissue or was combined with bone tissue, the need for another flap, postoperative complications, treatment with radiotherapy (RT), the need for readaptation of the flap and pedicle section, implant rehabilitation and edentulism. Patients completed the Spanish versions of both questionnaires at a follow-up without any influence on their responses to minimize measurement bias.

### 2.4. Statistical Analysis

A comparison method based on known comparative groups was performed, due to the absence of a gold standard [9]. Scores of the QLQ-C30 and QLQ-H&N35 questionnaires according to different sociodemographic and clinical parameters and lifestyle-related issues, were compared. Quantitative values were expressed as mean ± standard deviation (D.S) or median and interquartile range as well as total range, while qualitative variables were reported as frequencies and percentages. The Kruskal–Wallis and Mann–Whitney tests were used to compare differences between groups of quantitative variables. The statistical analysis was performed using the software SPSS 25.0. (IBM Corp. in Armonk, NY, USA). A two-tailed *p*-value of lower than 0.05 was considered statistically significant.

## 3. Results

A total of 39 patients with a mean age of 61.23 ± 15.80 years at the time of surgery (48.7% men, 51.3% women) were included in the study. Sociodemographic and clinical characteristics are summarized in Table 1.

Thirty-three were diagnosed with oncological disease (84.6%). A total of 81.8% corresponded to squamous cell carcinoma, 6% to other malignant tumors, such as embryonal rhabdomyosarcoma and polymorphous adenocarcinoma, and 12.1% to benign lesions, such as pleomorphic adenoma, giant cell granuloma and ossifying fibroma. Oncologic patients underwent resection with clear margins and neck dissection when indicated. Six patients presented (15.38%) orosinusal communication and underwent extensive debridement. All patients were immediately reconstructed with the buccinator flap.

In terms of tumor stage, according to AJCC guidelines, the majority of patients were stage I (72.4%). The location of the defect varied, with the most frequent being the tongue (33.3%). The area of the defect showed a mean size of 9.2 ± 4.9 cm^2^. A soft-tissue defect was reconstructed in 64.10% of patients, while in 35.9% of patients the reconstructed defect included soft tissue and bone. In 66.7% of the patients, no other flaps were necessary to perform the reconstruction of the defect. In patients in whom an additional flap was necessary, the Bichat fat pad flap was the most common technique used for reconstruction (92.3%). Fourteen patients (35.9%) required a second surgical procedure to readapt the flap.

The incidence of patients with postoperative radiotherapy (RT) was 38.5%, with a mean dose of 60Gy. Complications were reported in 38.46%: the most common were partial flap necrosis (six patients) and trismus (five patients), although only 32% required a subsequent procedure.

Sixteen (41%) patients were completely edentulous and twenty-one (53.9%) were partially edentulous, as a consequence of previous tooth loss or the need for extractions to avoid occlusal trauma to the pedicle. Osseointegrated implants have represented a significant advance in the reconstructive treatment of oncological patients. Because of this, patients can achieve an optimal reconstruction ensuring a fully esthetic and functional rehabilitation. Fourteen patients were rehabilitated with osseointegrated implants, and two patients were rehabilitated with mucosa-supported removable prostheses, because they declined dental-implant treatment. A total of 131 osseointegrated implants were placed. The implants were immediately placed in the same surgical procedure as the buccinator flap reconstruction. In edentulous patients, implants were placed in both the mandible and maxilla to achieve optimal functional reconstruction. In dentate patients who required extraction of the last molars to avoid flap damage, dental implants were placed at the same time as tooth extraction. In non-irradiated patients, prosthetic rehabilitation was performed 4 months after reconstructive surgery. In irradiated patients, dental rehabilitation was performed 8 months after the end of radiotherapy. The follow-up time was 2.9 years, with a range of 6.5 months to 6.2 years.

The questionnaires took between 15 and 20 min to complete. The scores of the QLQ-C30 and the specific module QLQ-H&N35 are shown in Table 2 and Table 3, respectively.

The median global-health-status score was 79.3, with an interquartile range between 75 and 91.7, with 100 being the highest score. Among the functional scales, emotional functioning was the lowest, with a median score of 76.9. The other items showed high scores, with interquartile ranges between 83.3 and 100. The lowest scores on the symptom scale, with 100 being the lowest score, were fatigue (17.7), pain (20) and insomnia (13.7). Despite this, all items had a mode of 0 and an interquartile range between 0 and 33.3 as a maximum.

As for the symptom items of the specific head-and-neck module (QLQ-H&N35), low scores were obtained, all being below 40 points. The most outstanding were dental problems (33.3), trismus (31.6) and dry mouth (37.6), followed by sticky saliva (24.8), problems with social eating (21.1), and pain (19.9). In addition, it is remarkable that almost half of the patients needed pain medication and that 89.7% used a temporary feeding tube in the first days after surgery.

As illustrated in Table 4 and Table 5, a comparison of the quality-of-life scales according to gender, shows that men showed more fatigue, pain and analgesic consumption than women. In terms of age, significant differences were found only in insomnia, and were more frequent in patients >60 years. No differences were found in smoking, alcohol consumption, aggressiveness and size of resection, dental rehabilitation or post-surgical follow-up.

Remarkably, the comparison of lesion location found no significant differences on the functional scale. Tumors located on the tongue and floor of the mouth scored high on functionality, although not as high as those on the palate or maxilla. In terms of global health and symptoms, although there were no significant differences, good scores were found in the maxillary and palatal regions. In terms of diagnosis, there were better scores for sequelae compared with the other two groups, although there were higher scores for functionality, global health and symptomatology in the recurrences compared with the primary diagnoses, although none of the comparisons were significant.

When compared in accordance with the AJCC staging guidelines (with the “No” category representing non-malignant tumors), it was observed that with increasing stage there was a decrease in functionality and health, and an increase in symptomatology. Despite the above, significant differences were only found in swallowing problems.

As a final comparison, quality of life was evaluated according to the application of RT. Worse functionality and increased symptomatology, such as difficulty in oral opening or thick saliva, were observed in the group treated with RT, but significant differences were found only in global health status, swallowing problems, dental problems, pain, and dry mouth.

## 4. Discussion

**Figure 1:**
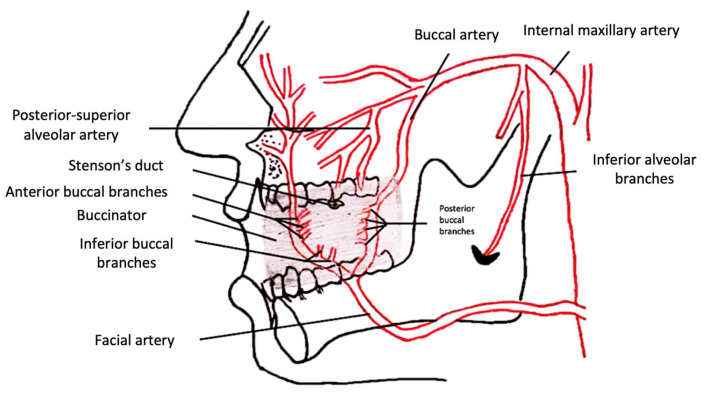
Vascular supply of the buccinator myomucosal flap.

The buccinator flap comprises mucosa, submucosa and muscle. It is limited superiorly by the Stenson’s duct, inferiorly by the mandibular vestibule, anteriorly by the oral commissure and posteriorly by the pterygomandibular raphe [4,10]. It has a wide vascular supply from both the facial artery and the buccal artery. Venous drainage is achieved through the submucosal venous plexus, the pterygoid venous plexus and the facial vein [1,11] (Figure 1). There are different options to harvest this flap. Rahpeyma [1] described in 2013 a classification based on its pedicle:

(a) Posterior buccinator myomucosal flap: based on the buccal artery (branch of the maxillary artery) and the posterior buccal artery (branch of the facial artery), which can be a pedicled flap or an island flap.
(b) Superior buccinator myomucosal flap: based on the angular artery (branch of the facial artery) with retrograde flow, which can be harvested as a pedicle flap or island flap.
(c) Inferior buccinator myomucosal flap: based on the facial artery with anterograde flow, which can be pedicled (facial artery myomucosal flap or FAMM flap) or dissected as an island flap (Zhao flap) [12].
(d) Anterior buccinator myomucosal flap: pedicled over the anterior buccal artery (branch of the facial artery).

The buccinator flap is very versatile, safe and reliable. It provides a wide arc of rotation for reconstruction of different locations such as the nasal cavity, palate, maxilla, tongue, floor of the mouth, mandible, oropharynx and lips [2,10]. In addition, it provides optimal thickness with a mucosa of similar color and texture to the rest of the intraoral soft tissues and the ability to secrete saliva, which allows for an excellent functional result [13,14]. Nevertheless, functional and aesthetic restoration remains a major challenge for head and neck surgeons [15]. It is not only the coverage of soft-tissue defects that is important, but also the functional outcome.

Restoration of functionality, esthetics and quality of life after oncologic surgery or intraoral sequelae are some of the main challenges of head and neck surgery. Optimal functionality requires good lingual mobility and adequate lip competence, to allow adequate swallowing, breathing, and speech, to perform basic daily needs [2].

Although the buccinator flap provides a limited width and requires a second-stage procedure in many patients, it offers many benefits: (1) it is a thin and pliable flap with a wide arc of rotation and length; (2) it is a myomucosal flap, ideal for reconstruction of mucosal defects because of the lack of hair; (3) it is a safe and reliable flap that allows postoperative radiotherapy; (4) it is easy to harvest; (10) it enables primary closure of the donor site under 3 cm^2^; (11) it can be harvested simultaneously with neck dissection; (12) previous neck dissection and radiation therapy are not contraindications for its use [12,13,14]; it can be superiorly or inferiorly pedicled, depending on the defect to be reconstructed [14]; it can be used as a reconstructive technique simultaneously with the immediate placement of osseointegrated implants for both aesthetic and functional rehabilitation.

The EORTC questionnaires, version 3.0 of the general module together with the H&N-35 module, have been implemented in this manuscript. These questionnaires are comprehensive, and their validity, internal consistency and reliability have been tested in large groups of patients [5,7,8,9]. However, it is a general head-and-neck-cancer quality-of-life scoring system, and may have some limitations for the evaluation of study patients.

The design of this study, being cross-sectional, implies that quality of life is measured once for each patient, which represents a limitation of the study. To assess changes over time, patients should be evaluated using a longitudinal study, in which quality of life is measured before, during and after treatment, for each patient. The scale scores of our patients are comparable to the results of previous studies [5,16,17].

Patients reported a good quality of life, showing values above 75 on the functioning scales and a global health status of 79.3, which indicates a good ability to perform daily activities, sociability and an overall high quality-of-life. The most significant symptoms were pain and fatigue in the general questionnaire. In the specific head-and-neck questionnaire, the patients reported more difficulties, which is consistent with previous studies [5,18]. Difficulty in oral opening, dry mouth and thick saliva obtained a higher mean score (<38). These problems were associated with the adverse effects of radiotherapy [19]. A total of 89.7% of the patients used a nasogastric feeding-tube in the postoperative period temporarily and, therefore, the quality of life was not influenced.

In general terms, the researchers highlight the absence of significant differences on the scales of physical, social, cognitive, emotional and role functioning, which allows us to suggest an adequate quality of life in spite of the buccinator flap reconstruction. The investigators also highlight the absence of differences in financial difficulties, presumably due to the fact that these patients experienced short hospital stays without significant impairment of their functionality. Furthermore, it is surprising that no differences were observed in sociability problems, both in social contact and social eating, nor in speech problems.

The gender of the patients is not an important parameter: the only anecdotal evidence obtained was that men presented greater pain than women. As for age, the differences obtained for insomnia seem to be more related to age itself than to the surgical procedure, since older people are more likely to have problems harmonizing their sleep.

The absence of differences in smoking and alcohol consumption is surprising to researchers, since both are the main oncologic risk factors for head and neck cancer. It is true that researchers find as an explanation the fact that most patients have tumors diagnosed in early stages, and this may influence the absence of significant differences. Something similar can be found with the follow-up period, and the size and the extension of the resection. Because they are tumors diagnosed in the early stages or they have benign pathologies, their resection is not usually extensive, and the average defect-area created is small (9.2 ± 4.9 cm^2^).

No differences in diagnosis were found, either. The investigators highlight the results in relation to the relapse group, which shows better functionality, better overall health and fewer symptoms than the other two groups. Presumably this is due to a sample-size bias (the relapse group consists of two patients), and therefore the results are not representative in this item.

In terms of AJCC stages, significant differences were found in swallowing. The results highlight worse symptoms from Stage III onwards, a stage which, according to clinical guidelines, implies the performance of adjuvant radiotherapy.

In terms of location, the maxilla and palate region showed better functional results than other locations. In the rest of the scales, although no significant differences were found, the maxilla and palate group obtained better results in global health and symptoms. The researchers explain these data by the fact that 71% of diagnoses in these regions were benign pathologies or non-oncological sequelae. In addition, it has been found that tumors located in the tongue and mandibular gingiva are more likely to develop swallowing and dry-mouth problems. This is due to the effect of radiotherapy and the possibility of these locations reaching stages that require adjuvant treatment, since 66.7% have undergone postoperative radiotherapy. It is surprising that the maxillary gingiva is not similar to the mandibular gingiva, although this could be explained by the low incidence of malignant neoplasms in the study sample.

As for the radiotherapy group, it is the only group in which overall health was significantly compromised. Surprisingly, no differences were found in the oral-opening and thick-saliva scales, due to a lack of statistical strength of the sample size. The highest symptom scores were fatigue, pain, dry mouth, oral-opening difficulties, dental problems, and swallowing. These side effects have been shown to be mainly due to radiotherapy and the consequences of irradiation on salivary glands, scar tissues, temporomandibular joint, teeth, and masticatory muscles. This leads us to conclude that buccinator-flap reconstruction surgery may result in greater morbidity and worse quality of life if adjuvant radiotherapy is subsequently considered.

Finally, no differences were found in patients rehabilitated with dental implants. The reason suggested by the researchers is that, despite the use of a flap that often requires tooth extraction and the immediate placement of dental implants, patients are rehabilitated with implant-supported prostheses in a short period of time, and most patients attach more importance to their oncological process than to the provisional absence of teeth.

## 5. Conclusions

Patients reconstructed with the buccinator myomucosal flap develop a good quality of life for all types of activities, and adequate functionality and aesthetics. The buccinator flap is an accurate and reliable reconstructive alternative for the reconstruction of medium-sized intraoral defects. It is a predictable flap that allows a like-for-like reconstruction of the oral cavity, with minimal morbidity. It is accepted that postoperative radiotherapy in itself is associated with a poorer quality of life, and that this type of surgery can lead to the impairment of several symptoms such as swallowing, oral opening and dry mouth. With this study the researchers conclude, using validated EORTC questionnaires, that patients reconstructed with the buccinator myomucosal flap obtain a good quality of life for all types of activities, and adequate functionality and aesthetics.

## Figures and Tables

**Table 1:** Description of the socio-demographic and clinical characteristics.

Variable	Category	Frequency	
		*n*	%
Age	≤60	20	51.3
	>60	19	48.7
Gender	Male	19	48.7
	Female	20	51.3
Smoking	No	20	51.3
	Yes	19	48.7
Alcohol consumption	No	30	76.9
	Yes	9	23
Diagnosis	Oncologic (Primary tumor)	31	79,5
	Oncologic (Recurrence)	2	5,1
	Iatrogenic sequelae	5	12.8
	Congenital sequelae	1	2.6
Tumor site	Tongue	13	33.3
	Mouth floor	9	23
	Lower jaw gingiva	3	7.7
	Upper jaw gingiva	6	15.4
	Palate	8	20.5
Resection	Soft tissue	25	64.1
	Combined tissue	14	35.9
Need for another flap	No	26	66.7
	Yes	13	33.3
	Bichat’s flaps	12	92.3
	Other	1	7.7
Stage (AJCC)	None	10	25.6
	I	9	23
	II	12	30.8
	III	6	15.4
	IVa	1	2.6
	IVb	1	2.6
Radiotherapy	No	24	61.5
	Yes	15	38.5
Complications	None	24	61.5
	Partial necrosis	6	15.4
	Complete necrosis	1	2.6
	Trismus	5	12.8
	Infection/dehiscence	2	5.1
	Neuropatic pain	1	2.6
Edentulism	Partial	21	53.9
	Complete	16	41
	No	2	5.1
Dental rehabilitation	No	12	30.8
	Yes *	16	41
	In process	11	28.2
Follow-up	≤3	23	59
	>3	16	41

* Two patients were rehabilitated with removable mucosa-supported prostheses (without osseointegrated implants).

**Table 2:** Total scores for QLQ-C30 questionnaire (Version 3.0).

QLQ-C30 Scale Name	Mean Score	SD	Median Score	IQR	Range
Functional scales					
Physical function	78.6	21	83.3	(66.7–100)	(33.3–100.00)
Role function	85	23.5	100.	(83.3–100)	(0–100)
Emotional function	76.9	23	83.3	(66.7–91.7)	(0–100)
Cognitive function	90.2	21.9	100	(83.3–100)	(0–100)
Social function	85.5	27.4	100	(83.3–100)	(0–100)
Symptom scales/items					
Fatigue	17.7	16.7	11.1	(0–22)	(0–55.6)
Nausea and vomiting	2.1	7.8	0	(0–0)	(0–33.3)
Pain	20.1	19.6	16.7	(0–33.3)	(0–66.7)
Dyspnea	11.1	20.7	0	(0–33.3)	(0–66.7)
Insomnia	13.7	21.2	0	(0–33.3)	(0–66.7)
Appetite loss	8.6	18.3	0	(0–0)	(0–66.7)
Constipation	12	24.8	0	(0–33.3)	(0–100)
Diarrhea	3.4	10.3	0	(0–0)	(0–33.3)
Financial difficulties	9.4	22.9	0	(0–0)	(0–100)
Global health status/qol					
Global health status	79.3	19.8	83.3	(75–91.7)	(16.7–100)

Abbreviations: QLQ = quality of life; SD = standard deviation; IQR = interquartile range.

**Table 3:** Total scores for QLQ-H&N35 questionnaire.

Scale Name	Mean Score	SD	Median Score	IQR	Range
Symptom scales/items					
Pain	19.9	18.8	16.7	(8.3–25)	(0–75)
Swallowing	12.8	18.3	8.3	(0–25)	(0–75)
Teeth	33.3	34.2	33.3	(0–66.7)	(0–100)
Opening mouth	31.6	31.5	33.3	(0–66.7)	(0–100)
Dry mouth	376	38.4	33.3	(0–66.7)	(0–100)
Sticky saliva	24.8	29.3	0	(0–33.3)	(0–100)
Sense problems	9.8	23.5	0	(0–0)	(0–100)
Coughing	14.5	22.7	0	(0–33.3)	(0–100)
Feeling ill	8.6	19.8	0	(0–0)	(0–100)
Speech problems	14.5	19.1	11.1	(0–22.2)	(0–100)
Troubles with social eating	21.2	22.2	16.7	(0–33.3)	(0–100)
Troubles with social contact	10.6	19.2	0	(0–13.3)	(0–73.3)
Less sexuality	16.2	26.4	0	(0–33.3)	(0–100)
Pain killers	46.2	50.5	0	(0–100)	(0–100)
Nutritional supplements	5.1	22.4	0	(0–0)	(0–100)
Feeding tube	89.7	30.7	100	(100–100)	(0–100)
Weight loss	30.8	46.8	0	(0–100)	(0–100)
Weight gain	43.6	50.2	0	(0–100)	(0–100)

Abbreviations: QLQ = quality of life; SD = Standard deviation; IQR = interquartile range.

**Table 4:** Comparison of groups of the QLQ-C30 questionnaire.

Variable	Categories	QLQ-C30 Questionnaire					
		Functional Scale		Symptom Scale		Global Health Status	
		Score (Mean ± SD)	*p* Value	Score (Mean ± SD)	*p* Value	Score (Mean ± SD)	*p* Value
Gender	Male	80.6 ± 18.8	0.139	11.8 ± 11.1	0.513	77.6 ± 21.4	0.629
	Female	85.7 ± 19.1		10.2 ± 10.9		80.8 ± 18.6	
Age	≤60	82.1 ± 21.2	0.933	11.9 ± 11.5	0.855	77.5 ± 21.1	0.528
	>60	84.4 ± 16.6		10 ± 10.4		81.1 ± 18.6	
Smoker	Yes	80.6 ± 26.2	0.373	12.2 ± 19.7	0.537	82.1 ± 15.6	0.403
	No	85.8 ± 20.5		9.6 ± 15.7		76.3 ± 23.5	
Alcohol consumption	Yes	80.4 ± 26.5	0.557	12.8 ± 19.3	0.34	79.4 ± 18	0.741
	No	84.1 ± 22.4		10.3 ± 16.8		78.7 ± 26.1	
Diagnosis	Primary tumour	80.3 ± 20	0.030	12.6 ± 11.7	0.218	76.9 ± 20.9	0.250
	Recurrence	92.5 ± 0.7		5 ± 1.4		83.3 ± 11.8	
	Sequelae	95.3 ± 7.6		4.7 ± 3.3		90.3 ± 11.1	
Tumour site	Tongue	80.2 ± 21.9	0.077	10.4 ± 11	0.302	74.4 ± 14.6	0.081
	Floor of the mouth	75.1 ± 24.2		15.2 ± 11.2		71.3 ± 33.1	
	Lower jaw gingiva	78.3 ± 11.6		18.3 ± 21.4		77.8 ± 9.6	
	Upper jaw gingiva	91.5 ± 4.6		6.5 ± 5.4		87.5 ± 7	
	Palate	93 ± 10.3		7.5 ± 8.1		90.6 ± 10.4	
Defect Size	<5 cm	84.2 ± 19	0.713	13 ± 21.1	0.585	88.5 ± 13.3	0.426
	5–10 cm	78.8 ± 30.1		11.2 ± 16.5		72.8 ± 27.7	
	10–15 cm	84.9 ± 20.3		11.7 ± 20.1		81.82 ± 11.1	
	>15 cm	91.7 ± 10.9		5.1 ± 5.7		78.3 ± 7.5	
Stage (AJCC)	No malignant	93.2 ± 9.5	0.144	5.7 ± 7.4	0.260	89.2 ± 9.7	0.217
	I	76.8 ± 2.4		11.6 ± 8.8		72.2 ± 28	
	II	78.3 ± 22.1		14.3 ± 12.4		79.2 ± 22.9	
	III	82.8 ± 12.1		12.2 ± 15.2		76.4 ± 8.2	
	IVA	93		4		66.7	
	IVB	93		4		75	
RT	Yes	81.1 ± 17.7	0.139	11.3 ± 10.6	0.409	75.6 ± 14.6	0.034
	No	84.6 ± 19.8		10.8 ± 11.3		81.6 ± 22.4	
Dental rehabilitation	Yes	85.5 ± 19.3	0.650	10.2 ± 17.7	0.420	85.4 ± 12	0.256
	No	81.6 ± 26.9		11 ± 16.9		74.6 ± 24.1	
Follow-up	</=3 years	81.1 ± 26.5	0.629	10.7 ± 17	0.690	75.4 ± 23.5	0.270
	>3 years	86.4 ± 18.6		11.2 ± 18.9		84.9 ± 11.1	

Abbreviations: QLQ = quality of life; H&N = Head and neck; SD = Standard deviation; RT = Radiotherapy.

**Table 5:** Comparison of groups of QLQ-H&N35 questionnaire.

Variable	Categories	QLQ-H&N35 QUESTIONNAIRE													
		Pain		Swallowing		Teeth		Opening Mouth		Dry Mouth		Sticky Saliva		Pain Killers	
		Score (M ± SD)	*p* Value	Score (M ± SD)	*p* Value	Score (M ± SD)	*p* Value	Score M ± SD)	*p* value	Score (M ± SD)	*p* Value	Score (M ± SD)	*p* Value	Score (M ± SD)	*p* Value
Gender	Male	26.3 ± 18.9	0.009	12.7 ± 19.1	0.722	33.3 ± 33.3	0.917	35.1 ± 34.2	0.572	36.8 ± 39.9	0.858	28.1 ± 31.9	0.560	73.7 ± 45.2	0.001
	Female	13.8 ± 16.9		12.9 ± 18		33.3 ± 35.9		28.3 ± 29.2		38.3 ± 37.9		21.7 ± 27.1		20 ± 41	
Age	≤60	19.2 ± 18	0.558	10.4 ± 18.3	0.219	28.3 ± 31.1	0.405	33.3 ± 30.6	0.647	46.7 ± 28.1	0.141	28.3 ± 24.8	0.209	5 ± 22.4	0.435
	>60	20.6 ± 20.1		15.4 ± 19.5		38.6 ± 37.3		29.8 ± 33.1		28.1 ± 37.3		21.1 ± 33.7		52.6 ± 51.3	
Smoker	Yes	20.4 ± 17.6	0.585	12.9 ± 18	0.847	35 ± 33.3	0.645	28.3 ± 32.9	0.396	38.3 ± 43.6	0.976	25 ± 30.4	0.988	55 ± 51	0.262
	No	19.3 ± 20.4		12.7 ± 19.1		31.6 ± 36		35.1 ± 30.4		36.9 ± 33.1		24.6 ± 29.1		36.8 ± 49.6	
Alcohol consumption	Yes	20 ± 17.7	0.670	14.4 ± 20.2	0.574	33.3 ± 35	0.916	32.2 ± 30.9	0.737	38.9 ± 39.2	0.738	27.8 ± 29.1	0.173	50 ± 50.9	0.385
	No	19.4 ± 23.2		7.4 ± 8.8		33.3 ± 33.3		29.6 ± 35.1		33.3 ± 37.3		14.8 ± 29.4		33.3 ± 50	
Diagnosis	Primary tumour	21.8 ± 20.3	0.45	13.2 ± 16.4	0.523	33.3 ± 33.3	0.983	34.4 ± 32.8	0.587	40.9 ± 30.2	0.518	25.8 ± 29.5	0.719	51.6 ± 50.8	0.298
	Recurrence	8.3		4.2 ± 5.9		33.3 ± 47.1		16.7 ± 23.8		33.3 ± 47.1		33.3 ± 47.1		0	
	Sequelae	13.9 ± 10.1		13.9 ± 30.1		33.3 ± 42.2		22.2 ± 27.2		22.2 ± 34.4		16.7 ± 27.9		33.3 ± 51.6	
Tumour site	Tongue	18.6 ± 21	0.390	19.9 ± 18.2	0.089	35.9 ± 34.6	0.879	38.5 ± 32.9	0.664	61.5 ± 35.6	0.071	30.8 ± 28.7	0.546	30.8 ± 48	0.252
	Floor of the mouth	24.1 ± 23.7		6.5 ± 9.1		33.3 ± 33.3		33.3 ± 33.3		3.3 ± 37.3		22.2 ± 28.7		55.6 ± 52.7	
	Lower jaw gingiva	38.9 ± 21		19.4 ± 26.8		44.4 ± 50.9		44.4 ± 50.9		33.3 ± 57.7		44.4 ± 50.9		100	
	Upper jaw gingiva	12.5 ± 4.6		2.8 ± 4.3		33.3 ± 29.8		16.7 ± 18.3		22.2 ± 27.2		22.2 ± 27.2		33.3 ± 51.6	
	Palate	15.6 ± 11.3		13.5 ± 26.3		25 ± 38.8		25 ± 29.6		16.7 ± 30.9		12.5 ± 24.8		50 ± 53.5	
Defect Size	<5 cm	20.8 ± 13.4	0.143	14.6 ± 17.7	0.565	29.2 ± 37.5	0.836	33.3 ± 35.6	0.693	25 ± 38.8	0.651	16.7 ± 35.6	0.586	75 ± 46.3	0.243
	5–10 cm	28.3 ± 24.8		14.4 ± 24.7		33.3 ± 39.8		40 ± 38.2		37.8 ± 35.3		28.9 ± 30.5		40 ± 50.7	
	10–15 cm	12.9 ± 10.1		7.6 ± 10.2		36.4 ± 23.4		24.2 ± 21.6		39.4 ± 38.9		27.3 ± 25		45.5 ± 52.2	
	>15 cm	8.3 ± 8.3		16.7 ± 11.8		33.3 ± 40.8		20 ± 18.3		53.6 ± 50.6		20 ± 29.8		20 ± 44.7	
Stage (AJCC)	No malignant	15 ± 8.6	0.613	8.3 ± 23.6	0.040	30 ± 36.7	0.581	30 ± 29.2	0.552	20 ± 28.1	0.079	20 ± 23.3	0.395	40 ± 51.6	0.816
	I	29.6 ± 21.7		13 ± 21.7		33.3 ± 37.3		33.3 ± 33.3		29.6 ± 38.9		22.2 ± 28.9		55.6 ± 52.7	
	II	18.1 ± 20.4		9 ± 10.9		22.8 ± 27.8		25 ± 32.2		33.3 ± 34.8		16.7 ± 26.6		50 ± 52.2	
	III	19.4 ± 25.1		26.4 ± 14.4		55.6 ± 40.4		50 ± 35		72.2 ± 39		44.4 ± 40.4		50 ± 54.8	
	IVA	16.7		25		33.3		33.3		100		33.3		0	
	IVB	8.3		8.3		0		0		66.7		66.7		0	
RT	Yes	26.7 ± 16.4	0.046	26.1 ± 22.5	0.000	46.7 ± 37.4	0.043	42.2 ± 32	0.103	53.3 ± 43.3	0.039	33.3 ± 35.6	0.052	60 ± 50.7	0.176
	No	16 ± 20.6		4.5 ± 7.8		25 ± 29.9		25 ± 29.9		27.8 ± 32.1		19.4 ± 23.9		37.5 ± 49.5	
Dental rehabilitation	Yes	15.1 ± 14.3	0.259	14.6 ± 20.1	0.606	33.3 ± 32.3	0.872	25 ± 25.8	0.362	33.3 ± 34.4	0.593	18.8 ± 27.1	0.292	43.8 ± 51.2	0.817
	No	23.8 ± 21.8		12.3 ± 18		36.5 ± 36.4		35.5 ± 34.8		41.3 ± 42		28.6 ± 30.3		47.6 ± 51.2	
Follow-up	</=3 years	20.7 ± 19.8	0.849	16.3 ± 21.5	0.278	37.7 ± 39.3	0.576	37.7 ± 35.3	0.221	44.9 ± 37.1	0.123	27.5 ± 32.8	0.663	39.1 ± 60	0.298
	>3 years	18.8 ± 17.9		7.8 ± 11.2		27.1 ± 25		22.9 ± 23.5		27.1 ± 38.9		20.8 ± 24		56.3 ± 51.2	

Abbreviations: QLQ = quality of life; H&N = Head and neck; SD = Standard deviation; RT = Radiotherap; M = Mean.

## Data Availability

The data presented in this study are available on request from the corresponding author. The data are not publicly available, due to data-protection regulations.
